# Heavy‐Textured Rhizosphere Soils Enhance Microbial Nitrogen Fixation in a Desert Shrub Ecosystem

**DOI:** 10.1002/ece3.71210

**Published:** 2025-04-09

**Authors:** Chenhua Li, Ran Liu, Lisong Tang, Li Jiang

**Affiliations:** ^1^ State Key Laboratory of Ecological Safety and Sustainable Development in Arid Lands Xinjiang Institute of Ecology and Geography, Chinese Academy of Sciences Urumqi Xinjiang China; ^2^ Fukang Station of Desert Ecology Chinese Academy of Sciences Fukang Xinjiang China; ^3^ University of Chinese Academy of Sciences Beijing China

**Keywords:** desert regions, microbial community, nitrogen (N) fixation, rhizosphere, soil texture

## Abstract

Understanding the relationships between nitrogen (N) fixation and soil texture and between soil and plants is important for the management of desert regions. The aim of this study is to compare the responses of N_2_ fixation and microbial communities to N deficiency in two soils with contrasting textures from the same desert region. We investigated heavy‐textured soils dominated by *Reaumuria soongorica* shrubs and sandy soils dominated by *Haloxylon ammodendron* shrubs in the Gurbantünggüt Desert. We induced a N‐deficient environment by introducing excess glucose into the soils and then conducted closed soil cultures using ^15^N‐labeled N_2_ and compared them with glucose‐free treatments. The driving mechanism of N fixation in soil has also been estimated. Analysis of stable isotope (^15^N) revealed significant N fixation throughout the heavy‐textured soil profiles (0–60 cm), particularly in the rhizosphere. The sandy soil had minimal N fixation and only in the bulk topsoil (0–20 cm). Redundancy analysis showed that microbial community shifts corresponded to dynamics of N_2_ fixation and were closely linked to soil texture and rhizosphere. Moreover, N_2_ fixation exhibited significant and positive correlations with several soil properties (e.g., available N, nitrate‐N, organic carbon, clay content), microbial activity, *nifH* gene abundance, and the relative abundance of certain microbial taxa (e.g., *Gillisia*, *Salinimicrobium*, and *Paraliobacillus*). Heavy‐textured rhizosphere soils with higher levels of organic carbon and nutrients displayed the greatest tendency for microbial N_2_ fixation under N stress. These findings suggest that heavy‐textured soils are probably more closely related to plants than sandy soils, from the perspective of supplying available nutrients and specific microbial communities to desert shrubs. This study evaluates the interrelationships among soil nutrients, rhizosphere, and microbial differentiation under N stress and explores a potential driver of free‐living N fixation in desert regions.

## Graphical abstract

N_2_ fixation occurred in a heavy‐textured soil profile (0–60 cm), especially in rhizospheres, and the sandy soil had slight N_2_ fixation only in the bulk topsoil (0–20 cm). Available N, nitrate‐N, SOC, clay content, microbial activity, nifH gene abundance, and the relative abundance of certain microbial taxa were the main drivers of N_2_ fixation in soils.

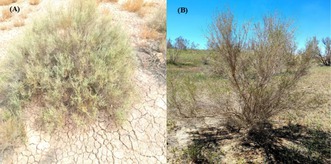

## 1 Introduction

In desert ecosystems, water is the most significant limitation on the growth of plants (Zou et al. 2010). Understanding plant‐water relationships has been a primary focus of researchers investigating ecosystem stability in deserts (Dai et al. 2015). Nevertheless, it is important to recognize that desert soils are inherently nutrient‐poor, and plant growth is frequently constrained by limited nutrient availability (Gallardo and Schlesinger 1992). Desert plants, particularly shrubs, seem to maintain rigid internal cycles by readily absorbing and recycling nutrients from the rhizosphere (Soussi et al. 2016). This phenomenon creates localized ‘fertility islands’ and results from the spatial variability of soil properties, exerting substantial impacts on ecosystem processes (Davies et al. 2023).

Nitrogen (N) is an indispensable element for the growth of all living organisms on Earth and is the primary limiting nutrient for desert plants (Huang et al. 2018). In natural ecosystems, N‐fixing microorganisms can convert atmospheric molecular N_2_ into ammonia, which can be directly absorbed by plants. Desert shrubs can survive for years in nutrient‐poor environments, and the truth is that root‐associated microbiota contribute to such adaptations (Mukhtar et al. 2021). Although soil microbial biomass is generally low in desert regions, the microorganisms remain highly active and play an important role in promoting plant health (Köberl et al. 2011). Recent research has suggested that soil microbial diversity may exert a more significant impact on ecosystem function and stability in desert regions when compared with plant diversity (Hu et al. 2021). Considering these findings, biological N_2_ fixation in soils is an important pathway for N supply under most conditions, particularly in desert regions. Long‐term cumulative productivity in desert plants, especially perennial shrubs, is likely to be constrained without a sustainable supply of N.

Nitrogen‐fixing microorganisms demonstrate their ability to fix N effectively in environments where carbohydrates are abundant and nitrogen compounds are lacking (Vitousek and Hobbie 2000). Additions of excess labile carbon (C) (usually D‐glucose or D‐mannitol at 0.5%–2.0%) can trigger the decomposition of soil organic matter by microorganisms, leading to a N‐deficient environment that promotes biological N fixation (Stowers 1985; Li et al. 1992; Hara et al. 2009). Moreover, research has shown that invasive species in an arid grassland can reduce available nitrogen for microbial activity by changing litter biomass and quality (i.e., increased C:N and lignin:N ratios), which, in turn, stimulates microbial nitrogen fixation (Evans et al. 2001). Such carbon‐nitrogen interactions in soils can further promote soil nitrogen storage (Perroni‐Ventura et al. 2010). However, information on nutrient availability and microbial N fixation in desert soils remains limited and fragmented, leaving uncertainties about the response of desert soils to the addition of exogenous carbon and its impact on N fixation.

Nitrogen‐fixing bacteria thrive in soil with rich organic matter and alkalinity. The ratios of exogenous organic carbon to nitrogen, soil nitrogen content, and salinity are closely related to microbial nitrogen fixation (Delgado et al. 1994; Juraeva et al. 2006). Soil texture significantly influences soil properties, which are closely related to the fixation capacity and leaching rate of water and nutrients, and microbial dynamics in soils (Rodríguez et al. 2009; Sugihara et al. 2010). Therefore, soil texture will inevitably affect N fixation by altering soil properties and microorganisms (Bicharanloo et al. 2022). This study focuses on two representative soils with contrasting textures, heavy textured and sandy, in the Gurbantünggüt Desert, Central Asia. For each soil, the bulk and rhizosphere soils of a dominant shrub species were selected (*Reaumuria soongorica* for heavy‐textured soils and *Haloxylon ammodendron* for sandy soils). In this region, desert farming is the primary type of land use. As a result, the selected soils in this study are original soils of adjacent farmlands. As a green ecological barrier, these shrubs have been contributing to the sustainability of the farmland ecosystem (Li et al. 2019). Our findings will provide guidance for sustainable land management, ecosystem services, and ecological restoration in desert regions.

In this study, N fixation and microbial activity were assessed under conditions of excess glucose addition and soil culture experiments coupled with ^15^N isotope tracing analysis. Furthermore, we employed quantitative real‐time PCR (qPCR) and MiSeq amplicon sequencing to assess the variations in *nifH* gene abundance and bacterial communities under carbon addition in soils, as affected by soil texture and rhizosphere. In addition, the soil properties were also determined. The objective is to determine and compare the responses of N fixation and microbial communities to N deficiency induced by excessive carbon addition in both soils. We hypothesized that (1) there should be a sharp contrast between the responses of microbial nitrogen fixation in the two soils due to differences in soil nutrient properties and (2) microbial community variation will correspond to dynamics of N_2_ fixation and is closely linked to soil texture and rhizosphere.

## 2 Materials and Methods

### 2.1 Study Area

**FIGURE 1:**
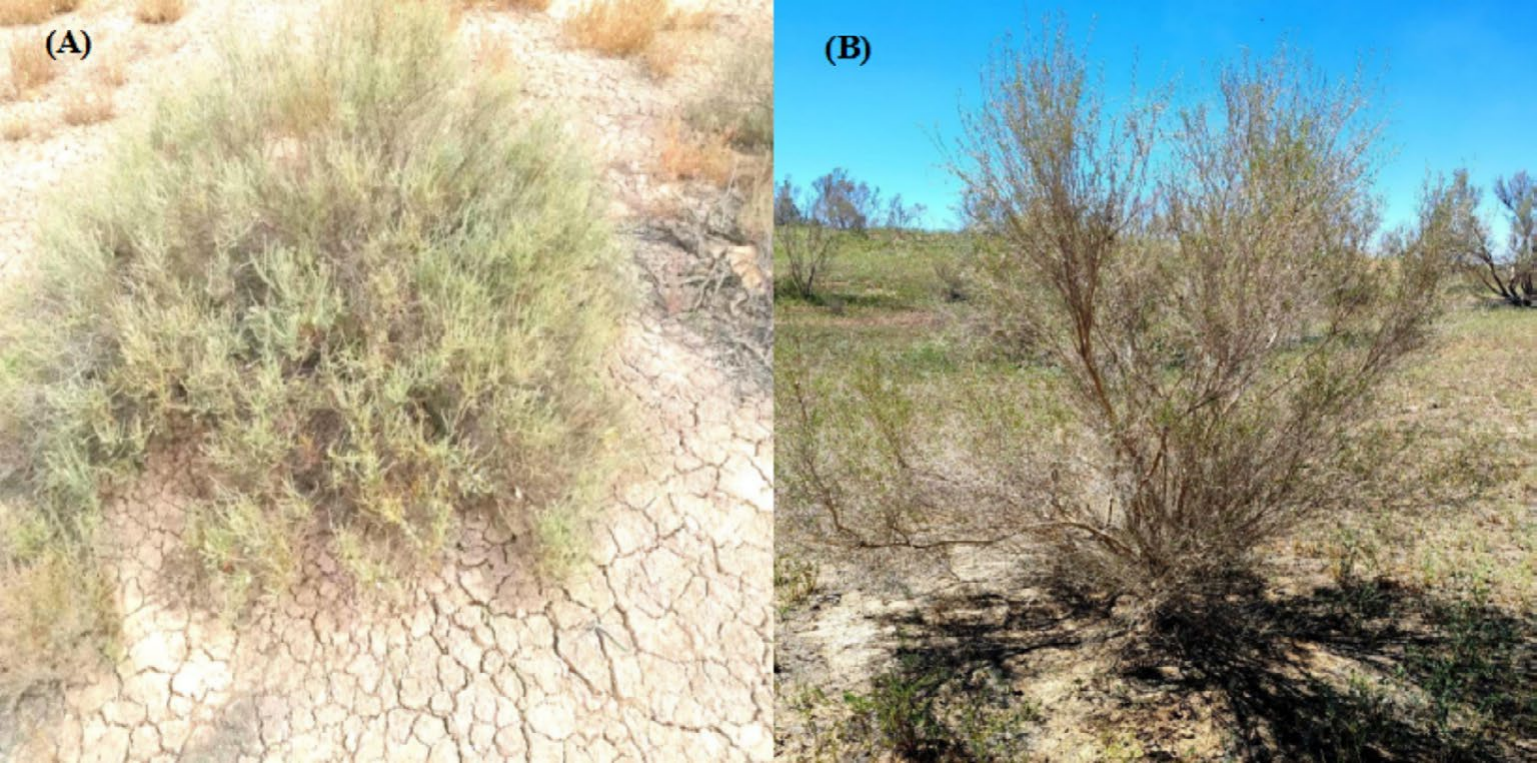
Heavy‐textured soils dominated by *Reaumuria soongorica* shrubs (A) and sandy soils dominated by *Haloxylon ammodendron* shrubs (B) in the Gurbantunggut Desert, center of the Eurasian continent.

Experiments were conducted near the Fukang Station of Desert Ecology, Chinese Academy of Sciences (44°17′  N, 87°56′  E, and 475 m a.s.l.). This region is a temperate desert with an arid continental climate that has a cold winter and dry hot summers. The annual mean temperature is 6.6°C, and the average monthly temperature ranges from −19.4°C in January to 25.8°C in July. Potential annual evaporation is 900 mm. The annual mean precipitation is 160 mm, of which 25% is generally snowfall. Snowfall events in the region occur primarily between January and March, with snow cover reaching a depth of 20–30 cm (Huang et al. 2018). The study focused on two representative soils situated along the southern edge of the Gurbantünggüt desert, approximately 8 km apart, experiencing identical climatic conditions without geomorphic changes. The soil types in these areas are classified as Torripsamments (sandy soil) under the soil order of Entisols, and Haplocalcids (heavy‐textured soil) under the soil order of Aridosols (USDA Soil Taxonomy) (Cao et al. 2016). *Haloxylon ammodendron* and *Reaumuria soongarica* are the dominant xerophytic shrub species in the sandy soil and in heavy‐textured soil, respectively (Figure 1). Both shrubs have well‐developed shallow root systems that efficiently absorb soil water and exhibit strong positive responses to increases in precipitation (Xu and Li 2006). The particle size distribution of the bulk and rhizosphere soils at depths of 0–60 cm for the two shrubs was presented in Table 1.

**TABLE 1:** Soil texture in bulk and rhizosphere soils of dominant shrub species in heavy‐textured and sandy soils.

Depth (cm)	Heavy‐textured bulk soil (%)			Heavy‐textured rhizosphere soil (%)		
	Clay	Silt	Sand	Clay	Silt	Sand
0–20^a^	7.6 a	68.5 a	23.9 b	7.0 a	65.6 a	27.4 b
20–40	6.3 a	66.8 a	26.9 b	6.9 a	62.0 a	31.1 b
40–60	5.9 a	63.9 a	30.2 b	6.0 a	59.0 a	35.0 b

Depth (cm)	Sandy bulk soil (%)			Sandy rhizosphere soil (%)		
	Clay	Silt	Sand	Clay	Silt	Sand
0–20	1.3 b	16.3 b	82.4 a	1.1 b	12.6 b	86.3 a
20–40	1.1 b	13.9 b	85.0 a	1.0 b	10.5 b	88.5 a
40–60	0.5 b	9.4 b	90.1 a	0.8 b	7.7 b	91.5 a

^a^ Treatment means within a given depth and particle size fraction followed by the same lowercase letter are not significantly different at *p* < 0.05.

### 2.2 Soil Sampling and Analysis

Soil sampling was conducted in early May 2022, as this time period corresponds to the highest soil moisture content and increased litter decomposition and rapid growth in desert plants due to snowmelt (Zhou et al. 2009; Thoms and Gleixner 2013). In each of the two soil areas, we randomly selected three plots (10 × 10 m). Three adult individual shrubs were also selected within each of these plots. The average height, basal diameters, and crown size were 200–210, 9.0–11.0, and 210–220 cm for *H. ammodendron*, respectively, and 70–80, 12.5–14.3, and 180–190 cm for *R. soongarica*. Three rhizosphere soil samples were collected randomly around each shrub, at depth intervals of 0–20, 20–40, and 40–60 cm, given that the shrub roots absorbing shallow soil water are mainly distributed in these layers. Samples from the same depth interval for each plot were mixed to obtain a representative rhizosphere soil sample. Additionally, bulk soil samples were collected from three random sampling points in the interplant for each of the plots.

The collected soil samples were divided into two parts. One part was air‐dried and passed through a 2 mm sieve for measuring soil properties. The other part was stored at −4°C until the soil culture experiment was carried out within 2 weeks. Soil physical and chemical properties, including soil particle sizes (soil texture), pH, electrical conductivity (EC), water content (SWC), organic carbon (SOC), total nitrogen (TN), total phosphorus (TP), available nitrogen (AN), and available phosphorus (AP), were determined using the methods described by Li et al. (2019). Additionally, the contents of extractable ammonium (${\mathrm{NH}}_{4}^{+}$‐N) and nitrate‐N (${\mathrm{NO}}_{3}^{-}$‐N) were determined using the AA3 flow injection analyzers (FIA SFA CFA).

### 2.3 Soil Culture Experiment

To determine the amount of carbon source addition and culture time, pre‐experiments were conducted using the same carbon source at varying amounts. Soil samples were divided into two parts in the laboratory: one for carbon addition experiments and the other as the control without carbon addition. The soil culture experiment was carried out in 120 mL serum bottles, each containing 6 g of soil (oven‐dry basis of field‐moist soil). In the carbon addition experiments, excess glucose was added to the soil which had a final concentration of 8 mg C g^−1^ dry soil. Each treatment was replicated three times. After adjusting to 60% water‐holding capacity, each soil portion was evenly spread at the bottom of each bottle. The serum bottles were sealed with rubber stoppers and reinforced with aluminum lids. They were pumped into a vacuum and then washed three times with high purity Argon. They were then injected with ^15^N‐N_2_ and O_2_ at a volume ratio of 2:3 and cultured at 25°C for 2 weeks. Gas from the bottles was collected with a sealed syringe, and the effluxes of CO_2_ were analyzed using a gas chromatography system (Agilent 7890, USA). Gas effluxes serve as indicators of microorganism respiration intensity (microbial activity), organic carbon mineralization, and nitrification reactions (Rousset et al. 2022). After freeze‐drying, the N isotopic values (δ^15^N) (atom %) of the soil were determined by element analyzer‐isotope mass spectrometry (EA‐IRMS) (Flash‐2000 Delta V Advantage, Germany). Additionally, the soil microbial community was analyzed.

### 2.4 *NifH* Gene Abundance and 16S rRNA Gene Pyrosequencing

Samples designated for microbial community analyses were frozen (−80°C) for soil DNA extraction and microbial gene analyses. Soil DNA was extracted from a minimum of 0.5 g of dry weight soil using a FastDNA spin kit (MP Biomedicals) and following the manufacturer's instructions. The *nifH* genes are used as typical marker genes for studying N‐fixing microbial communities (Zehr et al. 2003). In this study, the abundance of the *nifH* gene was quantified by determining the copy numbers by using quantitative real‐time PCR. Soil DNA extraction, quantitative PCR, and other relevant details followed the methods outlined in Li et al. (2023). Furthermore, N fixation in soils is found mainly in prokaryotes. In addition to typical molybdenum dinitride enzymes (mainly encoded by the *nifH* gene), there are other known alternative nitrogenases (Thiel and Pratte 2013). Therefore, the 16S rRNA sequencing was used in this study to comprehensively detect N‐fixing microbial communities.

The 16S rRNA gene pyrosequencing was performed on the HiSeq 2500 sequencing system (Illumina Inc.). To initiate the process, microbial DNA extracts from the soil were subjected to quality control measures and used as templates during PCR amplification in triplicate. For amplification of the V3–V4 hypervariable region of the 16S rRNA gene from bacteria, the 338f (5′‐ACTCCTACGGGAGGCAGCA‐3′) and 806r (5′‐GGACTACHVGGGTWTCTAAT‐3′) primers (McBain et al. 2003) were used and tagged with unique and sample‐specific barcodes. PCR reactions were conducted in a 50 μL mixture containing 50 ng of DNA, 10 μL of Q5 High‐Fidelity DNA Polymerase, 10 μL of High GC Enhancer, 10 μL of buffer, and 1.5 μL of each primer (10 μM). The PCR program was as follows: 95°C for 5.0 min; 30 cycles of 95°C for 1 min; 50°C for 1 min; 72°C for 1 min; 72°C for 7 min. The PCR products were purified, quantified, and homogenized to generate a sequencing library, which was then prepared for pyrosequencing analysis. Further details regarding pyrosequencing can be found in Li et al. (2023).

Using Flash v1.2.7, the base sequences of each sample were spliced by overlap to obtain the original sequence data. Next, the original sequences were filtered by Trimmomatic v0.33 to obtain high‐quality overlapping data. Finally, the effective sequences were obtained by identifying and removing chimeric sequences by using UCHIME v4.2. These high‐quality sequences were then clustered into operational taxonomic units (OTUs) at a 97% similarity level using the UCLUST algorithm in QIIME (version 1.8.0) (Edgar 2010). Taxonomic identity from each OTU was determined with the Ribosomal Database Project (RDP) Classifier (http://rdp.cme.msu.edu/). More relevant details can be found in Li et al. (2023). A total of 4,826,739 high‐quality sequences were obtained, and an average of 80,026 clean tags were generated per sample.

### 2.5 Data and Statistical Analyses

Statistical analyses were conducted using SPSS 20.0 for Windows (SPSS Inc.). Analysis of variance (ANOVA) and a Duncan's test were used to assess significant differences in soil physical and chemical properties among the bulk and rhizosphere soils of two dominant desert shrubs from two soil areas with distinct soil textures, after the data were checked for normality and homogeneity of variance. Moreover, the three‐way ANOVA with Tukey's test was performed to analyze the impacts of carbon addition and soil texture on the soil parameters (i.e., δ^15^N, CO_2_, and *nifH* gene abundance) and the relative abundance of the main bacterial groups within soil depths. The Pearson correlation was used to test the significance of correlations between selected parameters. Significance was *p* < 0.05.

Redundancy analysis (RDA) was applied to evaluate the relationship between the original soil properties and microbial community structure under N deficiency resulting from excessive carbon addition. This analysis allowed for a comprehensive assessment of microbial community shifts across soil texture, rhizosphere, and soil depth under N stress. Species data were square‐root transformed in the course of this analysis. The ordination plot was generated using CANOCO 5.0 for Windows. The effects of environmental variances, including pH, EC, SOC, TN, TP, AN, ${\mathrm{NH}}_{4}^{+}$‐N, ${\mathrm{NO}}_{3}^{-}$‐N, AP, and soil texture (clay, silt, and sand), were identified by the forward‐selection procedure with the Monte Carlo test (499 permutations).

## 3 Results

### 3.1 Soil Properties

Heavy‐textured soil exhibited significantly higher water content, EC, SOC, and nutrients (TN, TP, and AP) compared to sandy soil within the profile (*p* < 0.05) (Table 2). The contents of AN and ${\mathrm{NO}}_{3}^{-}$‐N in heavy‐textured bulk soil were also higher than in sandy bulk soil (*p* < 0.05). However, ${\mathrm{NH}}_{4}^{+}$‐N content and pH showed no significant difference among soils. Compared with the corresponding bulk soils, the content of SOC, TN, and AP substantially decreased, while the content of AN and ${\mathrm{NO}}_{3}^{-}$‐N significantly increased in the two rhizosphere soils with contrasting textures (*p* < 0.05). Furthermore, the water content in sandy rhizosphere soil was higher than that in sandy bulk soil (*p* < 0.05) and there was no significant difference between heavy‐textured bulk and rhizosphere soils.

**TABLE 2:** Soil water content (SWC), pH, electrical conductive (EC), total phosphorous (TP), available phosphorus (AP), soil organic carbon (SOC), total nitrogen (TN), available nitrogen (AN), ammonium nitrogen (${\mathrm{NH}}_{4}^{+}$‐N) and nitrate nitrogen (${\mathrm{NO}}_{3}^{-}$‐N) in bulk and rhizosphere soils of dominant shrub species in heavy‐textured and sandy soils.

Depth (cm)	SWC (%)	pH	EC (mS cm^−1^)	TP (g kg^−1^)	AP (mg kg^−1^)
Heavy‐textured bulk soil^a^					
0–20	5.9 b	9.1 b	7.8 a	1.0 a	27.4 a
20–40	9.9 a	8.6 d	6.2 a	0.7 a	13.6 a
40–60	8.3 a	8.9 b	6.3 a	0.7 a	5.5 a
Heavy‐textured rhizosphere soil					
0–20	6.9 a	8.3 c	3.6 b	1.1 a	18.2 b
20–40	7.1 b	9.6 bc	4.4 b	0.8 a	4.5 b
40–60	8.4 a	9.1 ab	5.4 b	0.7 a	5.4 a
Sandy bulk soil					
0–20	2.3 d	8.8 b	0.2 c	0.5 b	9.5 c
20–40	2.5 d	9.3 c	0.3 c	0.4 b	3.8 c
40–60	1.5 c	9.3 a	0.9 c	0.4 b	3.0 b
Sandy rhizosphere soil					
0–20	3.3 c	10.1 a	0.5 c	0.4 b	3.5 d
20–40	4.3 c	10.1 a	0.6 c	0.3 b	3.0 d
40–60	4.1 b	8.0 c	1.0 c	0.3 b	2.4 c

Depth (cm)	SOC (g kg^−1^)	TN (g kg^−1^)	AN (mg kg^−1^)	${\mathrm{NH}}_{4}^{+}$‐N (mg kg^−1^)	${\mathrm{NO}}_{3}^{-}$‐N (mg kg^−1^)
Heavy‐textured bulk soil					
0–20	4.5 a	0.58 a	28.5b	1.77 b	14.8 b
20–40	2.8 a	0.31 a	21.0 b	2.99 b	14.5 b
40–60	2.4 a	0.32 a	10.7 bc	2.62 bc	7.5 b
Heavy‐textured rhizosphere soil					
0–20	4.0 b	0.48 b	38.1 a	2.20 ab	24.8 a
20–40	2.1 b	0.29 a	25.9 a	3.75 a	19.5 a
40–60	1.9 b	0.25 b	18.4 a	3.66 a	13.7a
Sandy bulk soil					
0–20	1.7 c	0.23 c	24.1 c	2.46 a	6.2 c
20–40	0.8 c	0.09 b	13.8 d	2.26 bc	10.2 c
40–60	0.8 c	0.10 c	9.8 c	2.97 ab	5.0 c
Sandy rhizosphere soil					
0–20	1.0 d	0.15 d	25.3 bc	2.56 a	12.6 b
20–40	0.5 d	0.08 b	16.8 c	2.14 c	12.9 b
40–60	0.4 d	0.07 c	11.2 b	2.14 c	7.5 b

^a^ Treatment means within a given depth followed by the same lowercase letter are not significantly different at *p* < 0.05.

### 3.2 Changes in Soil N Isotopic Values (δ^15^N)

**FIGURE 2:**
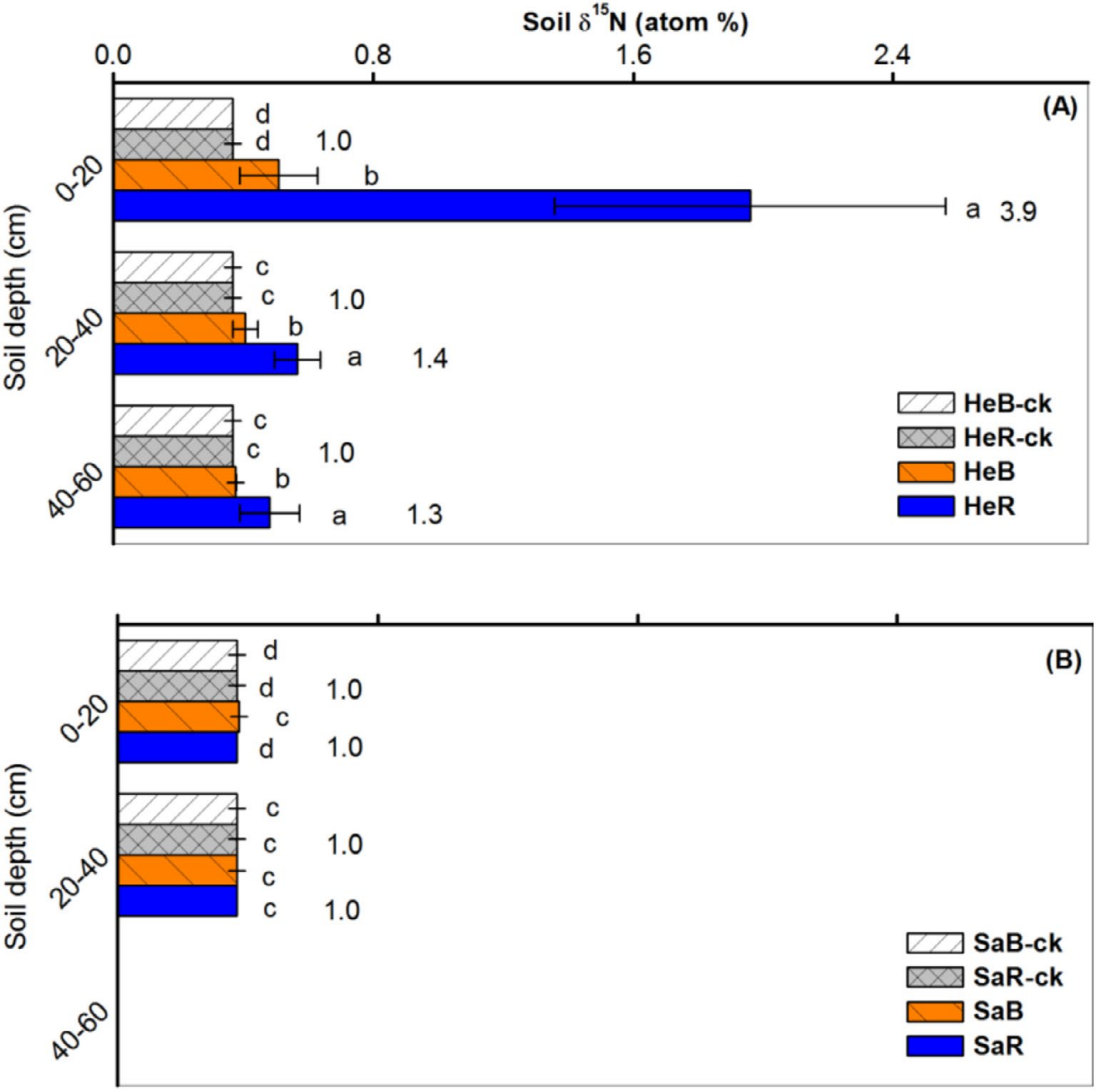
Responses of natural ^15^N abundance (δ^15^N) (mean + SE) to excessive glucose addition in bulk and rhizosphere soils of dominant shrub species in heavy‐textured (A) and sandy (B) soils. Different lower‐case letters indicate differences between treatment means within a given depth (*p* < 0.05). The numbers in the columns indicate rhizosphere effect (root/soil). HeB, Heavy‐textured bulk soils with excess glucose; HeB‐ck, Heavy‐textured bulk soils; HeR, Heavy‐textured rhizosphere soils with excess glucose; HeR‐ck, Heavy‐textured rhizosphere soils; SaB, Sandy bulk soils with excess glucose; SaB‐ck, Sandy bulk soils; SaR, Sandy rhizosphere soils with excess glucose; SaR‐ck, Sandy rhizosphere soils.

Both C addition and soil texture exerted significant impacts on soil δ^15^N (*p* < 0.001; Table S1). No significant difference in δ^15^N was observed among the controls (no carbon addition) in two soils (Figure 2). In the heavy‐textured soil, the treatments with excess carbon addition exhibited a substantial increase in δ^15^N throughout the entire soil profile (0–60 cm) compared to their corresponding controls (*p* < 0.05; Figure 2A). Notably, the values in rhizosphere soils exhibited significantly higher δ^15^N than bulk soils (*p* < 0.01), with the greatest rhizosphere effects in the topsoil (0–20 cm) (*p* < 0.001). In contrast, the sandy soil only exhibited a significant increase in δ^15^N in the bulk topsoil following the addition of excess glucose (Figure 2B).

### 3.3 Changes in Soil CO_2_ Efflux

**FIGURE 3:**
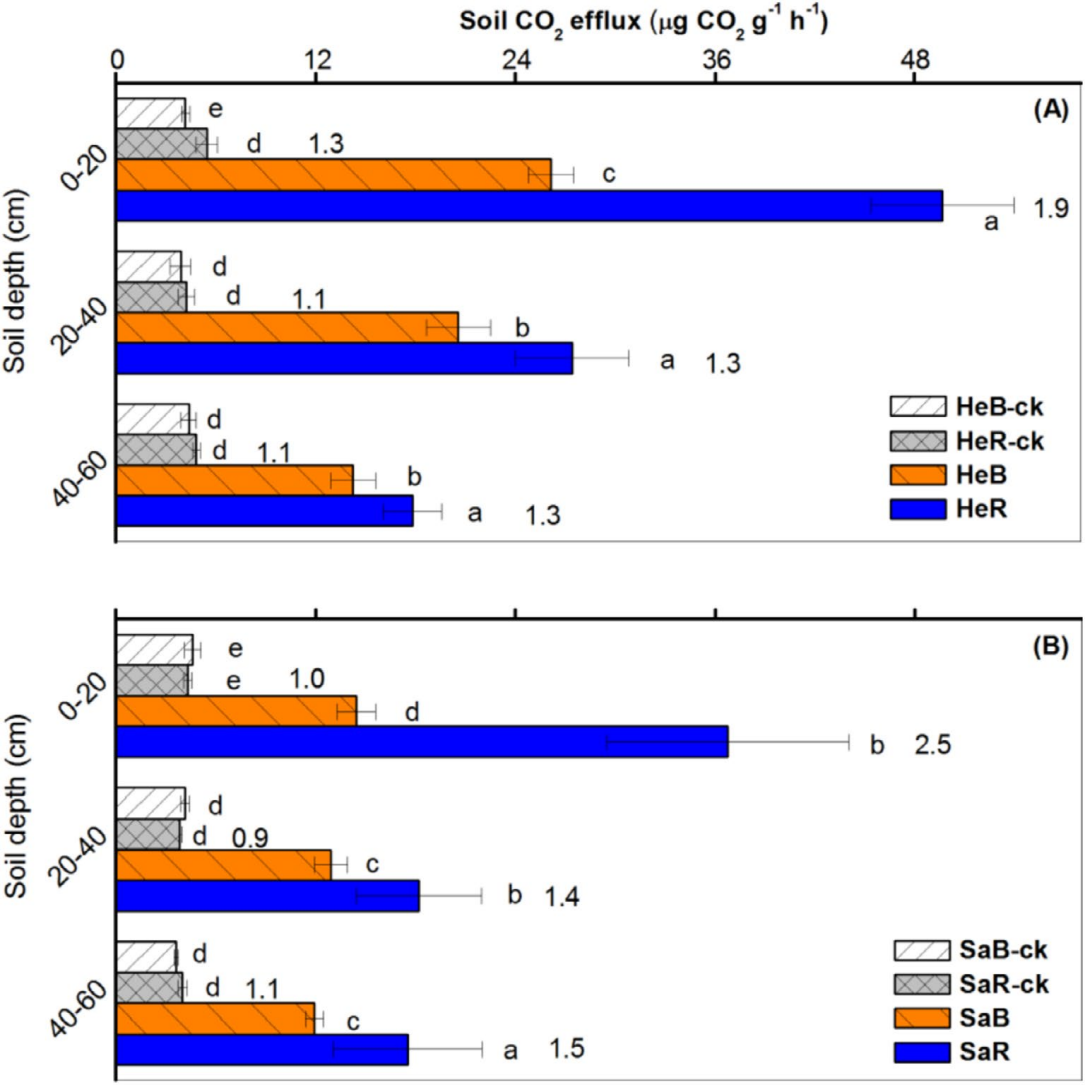
Responses of CO_2_ efflux (mean + SE) to excessive glucose addition in bulk and rhizosphere soils of dominant shrub species in heavy‐textured (A) and sandy (B) soils. Different lower‐case letters indicate differences between treatment means within a given depth (*p* < 0.05). The numbers in the columns indicate rhizosphere effect (root/soil). HeB, Heavy‐textured bulk soils with excess glucose; HeB‐ck, Heavy‐textured bulk soils; HeR, Heavy‐textured rhizosphere soils with excess glucose; HeR‐ck, Heavy‐textured rhizosphere soils; SaB, Sandy bulk soils with excess glucose; SaB‐ck, Sandy bulk soils; SaR, Sandy rhizosphere soils with excess glucose; SaR‐ck, Sandy rhizosphere soils.

Both carbon addition and soil texture had significant effects on soil CO_2_ efflux (*p* < 0.01; Table S1). Compared with the corresponding controls, the treatments with carbon addition resulted in a greater CO_2_ efflux in both soils. The highest CO_2_ efflux was observed in the topsoil (0–20 cm) of all treatments with carbon addition from the heavy‐textured soils (*p* < 0.05; Figure 3). Additionally, the rhizosphere effect on CO_2_ efflux from the treatments with C addition was significantly stronger than the controls (*p* < 0.05). Moreover, the CO_2_ efflux in heavy‐textured soils was significantly higher than in sandy soils throughout most of the soil profile (*p* < 0.05).

### 3.4 Changes in Soil Microbial Communities

For the *nifH* gene abundance, soil texture has a significant effect, but carbon addition has no significant effect (Table S1). Gene abundance in heavy‐textured soils was significantly higher than in sandy soils (Table 3). Excess carbon addition substantially increased gene abundance throughout the profile of heavy‐textured rhizosphere soils. However, the *nifH* gene abundance decreased significantly in sandy bulk soil after adding excess carbon, especially at 0–40 cm depths.

**TABLE 3:** Reponses of *nifH* gene abundance to excess glucose addition in bulk and rhizosphere soils of dominant shrub species in heavy‐textured and sandy soils.

Depth (cm)	Heavy‐textured soil (Copy number×10^3^ g^−1^)				Sandy soil (Copy numbers×10^3^ g^−1^)			
	HeB‐ck	HeR‐ck	HeB	HeR	SaB‐ck	SaR‐ck	SaB	SaR
0–20	16.2 d	26.4 b	20.3 c	29.0 a	10.5 e	4.6 g	8.1 f	7.5 f
20–40	12.1 b	7.6 c	7.1 c	16.2 a	6.2 d	3.4 e	4.5 e	3.3 e
40–60	10.2 a	4.9 c	5.3 c	6.9 b	3.8 d	1.5 e	3.2 d	2.0 e

*Note:* Treatment means within a given depth followed by the same lowercase letter are not significantly different at *p* < 0.05.

Abbreviations: HeB, Heavy‐textured bulk soils with excess glucose; HeB‐ck, Heavy‐textured bulk soils; HeR, Heavy‐textured rhizosphere soils with excess glucose; HeR‐ck, Heavy‐textured rhizosphere soils; SaB, Sandy bulk soils with excess glucose; SaB‐ck, Sandy bulk soils; SaR, Sandy rhizosphere soils with excess glucose; SaR‐ck, Sandy rhizosphere soils.

**FIGURE 4:**
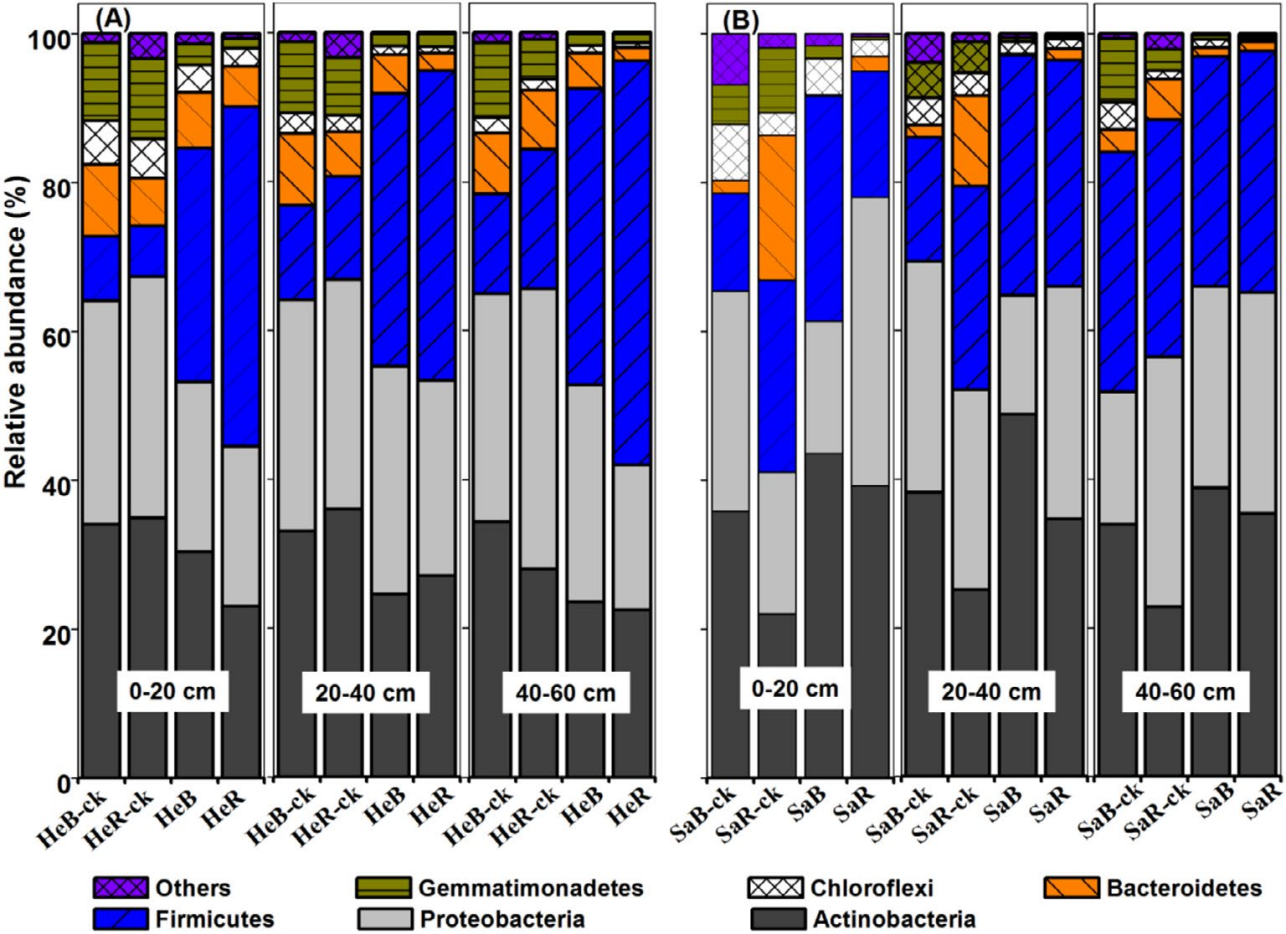
Responses of relative abundances of selected bacterial phyla to excessive glucose addition in bulk and rhizosphere soils of dominant shrub species in heavy‐textured (A) and sandy (B) soils. HeB, Heavy‐textured bulk soils with excess glucose; HeB‐ck, Heavy‐textured bulk soils; HeR, Heavy‐textured rhizosphere soils with excess glucose; HeR‐ck, Heavy‐textured rhizosphere soils; SaB, Sandy bulk soils with excess glucose; SaB‐ck, Sandy bulk soils; SaR, Sandy rhizosphere soils with excess glucose; SaR‐ck, Sandy rhizosphere soils.

*Actinobacteria* and *Proteobacteria* were the dominant phyla in both soils (Figure 4). The addition of excess glucose affected differently the relative abundance of some microorganisms in the two soils (*p* < 0.05). For example, after adding excess glucose, the relative abundance of *Actinobacteria* decreased (by 12%–52%) in heavy‐textured soil but increased (by 15%–78%) in sandy soil throughout the entire profile; the relative abundance of *Firmicutes* increased (by 189%–565%) in heavy‐textured soil with a positive rhizosphere effect, while the abundance had no obvious change in the whole profile of sandy soil.

**FIGURE 5:**
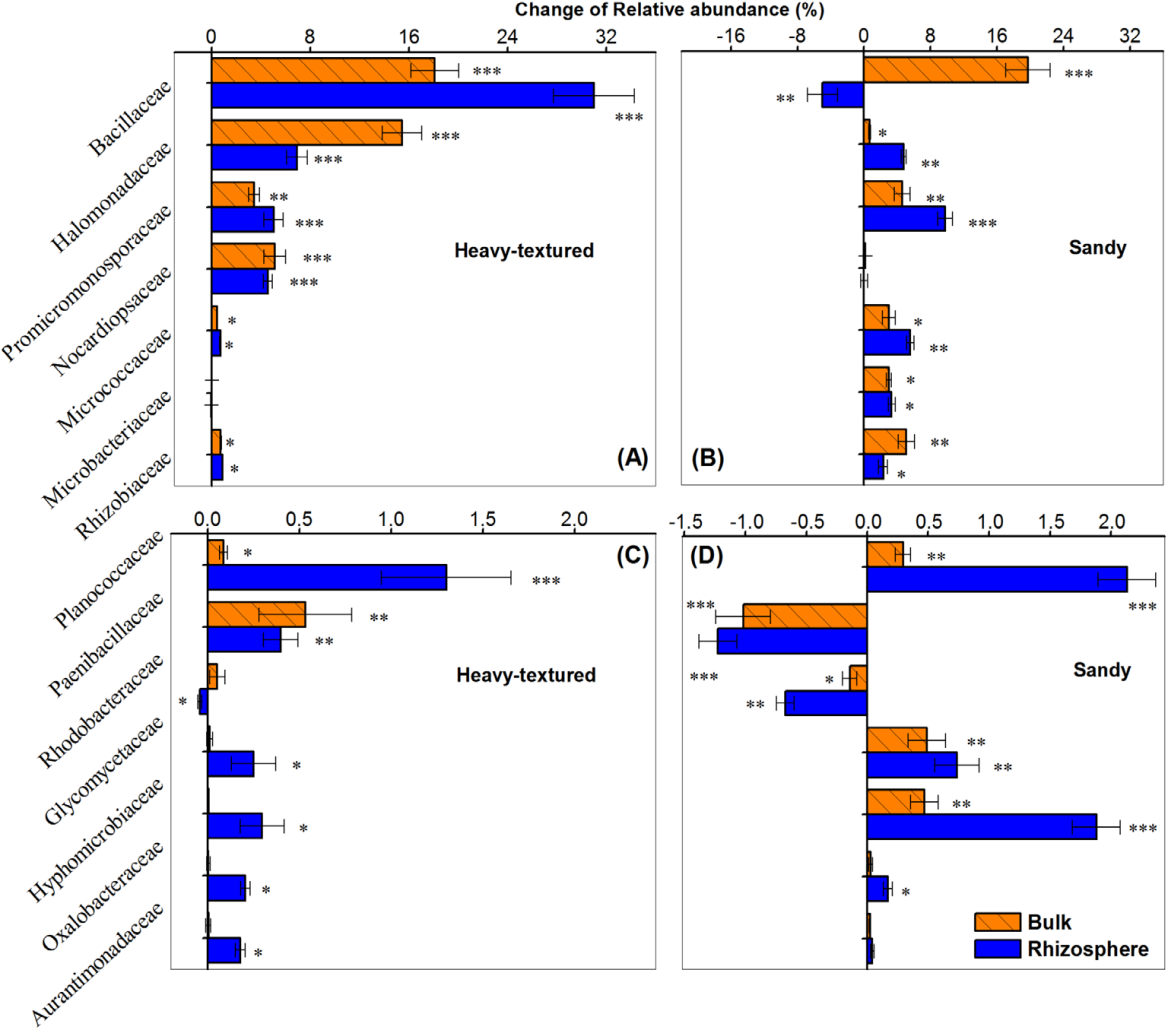
Fourteen bacterial families with significant changes in relative abundances (mean + SE) under excessive glucose addition at 0–60 cm depths in bulk and rhizosphere soils of dominant shrub species in heavy‐textured (A, C) and sandy (B, D) soils, compared to their corresponding controls. The stars indicate significant differences in abundance between treatments (***, *p* < 0.001; **, *p* < 0.01; *, *p* < 0.05).

For bacterial families, excess carbon addition significantly increased the relative abundance of Halomonadaceae, Promicromonosporaceae, Micrococcaceae, Rhizobiaceae, and Planococcaceae in both soils (*p* < 0.05; Figure 5). The relative abundance of Bacillaceae, Nocardiopsaceae, and Paenibacillaceae increased in heavy‐textured soil, and Microbacteriaceae, Glycomycetaceae, and Hyphomicrobiaceae increased in sandy soils. Positive rhizosphere effects were found on Promicromonosporaceae, Planococcaceae, Glycomycetaceae, Hyphomicrobiaceae, and Oxalobacteraceae in both soils (*p* < 0.05).

### 3.5 Correlation Analysis

**FIGURE 6:**
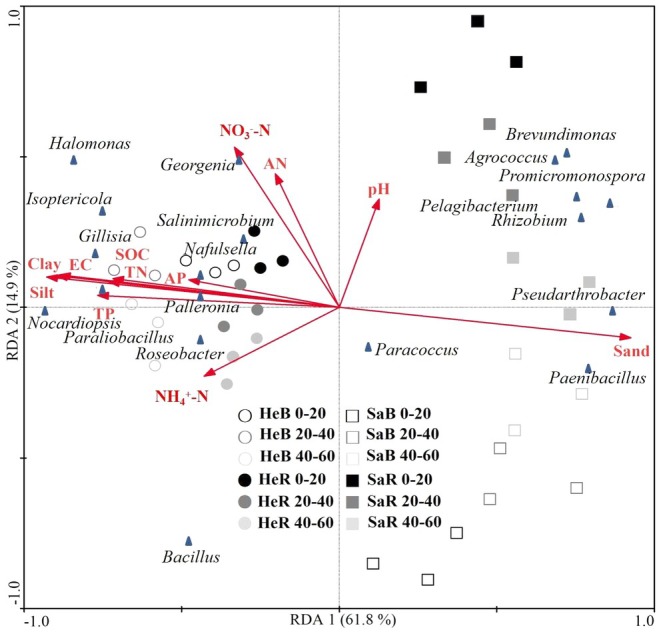
Ordination plots of the results from redundancy analysis (RDA) at 0–20, 20–40, 40–60 cm depths to explore the relationship between microbial community structure under excessive glucose addition and original soil properties, such as soil texture (clay, silt, and sand), soil pH, electric conductivity (EC), soil total nitrogen (TN) and available nitrogen (AN), ammonium nitrogen (${\mathrm{NH}}_{4}^{+}$–N), nitrate nitrogen (${\mathrm{NO}}_{3}^{-}$–N), total (TP) and available phosphorus (AP), soil organic carbon (SOC) for bulk and rhizosphere soils of dominant shrub species in heavy‐textured and sandy soils. HeB: Heavy‐textured bulk soils with excess glucose; HeR: Heavy‐textured rhizosphere soils with excess glucose; SaB: Sandy bulk soils with excess glucose; SaR: Sandy rhizosphere soils with excess glucose.

The RDA ordination plot exhibits that the heavy‐textured soils (including bulk and rhizosphere soil) were centered on areas with relatively high clay, silt, SOC, and nutrient content, while the sandy soils were centered along a gradient with higher sand and lower SOC and nutrient content (Figure 6). This showed a great differentiation of microbial community structure between the two soils under excessive carbon addition. The distribution of all treatments (soil depths, bulk, and rhizosphere) for the heavy‐textured soil was relatively concentrated, while the treatments for the sandy soil were distributed over a wide area in the RDA ordination plot. Thus, the microbial community structure was similar among the heavy‐textured soil treatments but relatively different among the sandy soil treatments.

According to the forward‐selection option in CANOCO (Table S2), soil texture, EC, SOC, and nutrient content (i.e., TN and AP) significantly affected the soil microbial community under excess carbon addition (*p* < 0.05). The order of influence was: silt> clay > sand > EC > SOC > TN > TP. Positive correlations were found between some soil properties (i.e., AN, ${\mathrm{NO}}_{3}^{-}$‐N, TP, SOC, TN, clay content) and soil δ^15^N under excess carbon addition. The relative abundance of certain microbial taxa (e.g., *Paraliobacillus*, *Palleronia*, *Isoptericola*, *Gillisia*, and *Salinimicrobium*), CO_2_ efflux, and *nifH* gene abundance exhibited positive correlations with soil δ^15^N (*p* < 0.05; Table 4). Soil CO_2_ efflux was positively and significantly correlated with some microbial groups (*Salinimicrobium*, *Gillisia*, *Isoptericola*, and *Georgenia*) and AN, ${\mathrm{NO}}_{3}^{-}$‐N content (*p* < 0.05).

**TABLE 4:** Correlation of original soil properties [i.e., pH, electrical conductivity (EC), total phosphorus (TP), available phosphorus (AP), organic carbon (SOC), total nitrogen (TN), available nitrogen (AN), ammonium nitrogen (${\mathrm{NH}}_{4}^{+}$–N), nitrate nitrogen (${\mathrm{NO}}_{3}^{-}$–N), soil texture (silt, clay, and sand)], CO_2_ effluxes, *nifH* gene abundance, and selected bacterial taxa with significant changes under excessive glucose addition with soil N isotopic values (δ^15^N) and CO_2_ efflux.

	δ^15^N		δ^15^N		δ^15^N		δ^15^N		δ^15^N
pH	0.246	AP	0.512	AN	0.771*	Clay	0.602*	CO_2_	0.678*
EC	0.239	SOC	0.646*	${\mathrm{NH}}_{4}^{+}$–N	−0.118	Silt	0.516	*nifH*	0.675*
TP	0.737*	TN	0.618*	${\mathrm{NO}}_{3}^{-}$–N	0.749*	Sand	−0.525		
*Salinimicrobium*		*Gillisia*		*Isoptericola*		*Palleronia*		*Paraliobacillus*	
	0.977**		0.905**		0.746*		0.638*		0.602*

	CO_2_		CO_2_		CO_2_		CO_2_		CO_2_
pH	−0.036	AP	0.440	AN	0.808*	Clay	0.460	*nifH*	−0.230
EC	0.148	SOC	0.521	${\mathrm{NH}}_{4}^{+}$–N	−0.031	Silt	0.361		
TP	0.585	TN	0.520	${\mathrm{NO}}_{3}^{-}$–N	0.730*	Sand	−0.471		
*Salinimicrobium*		*Gillisia*		*Isoptericola*		*Georgenia*			
	0.771*		0.709*		0.635*		0.601*		

*Note:* *p < 0.05; **p < 0.01.

## 4 Discussion

### 4.1 Responses of N_2_ Fixation to Excess Carbon Addition

This study confirms our hypothesis that the two soils exhibited completely different N fixation responses to excess glucose addition. First, significant N_2_ fixation was found throughout the heavy‐textured soil profile (0–60 cm), especially in rhizosphere soils. In contrast, the sandy soil had slight N_2_ fixation only in the bulk topsoil (0–20 cm) (Figure 2). Our results suggest that when the reduced carbon source is sufficient, biological N_2_ fixation is initiated in desert soil, but the response of N fixation varies with soil texture, rhizosphere, and soil depth.

In arid desert soils, N_2_‐fixation by biological soil crusts and free‐living, heterotrophic bacteria provides a large source of ecosystem N (Belnap 2002; Billings et al. 2003). Heterotrophic N_2_ fixation in bulk soils also significantly contributes to arid land ecosystem N (Strauss et al. 2012). Biological soil crusts contain key members such as cyanobacteria, eukaryotic algae, lichens, fungi, and other bacteria within the thin soil surface layer (Belnap 2002). Moreover, crusts are primarily distributed on bulk soils and are rarely found beneath shrub canopy in this desert area (Li et al. 2023). Hence, the most significant N_2_ fixation found in heavy‐textured rhizosphere soils in this study suggests that the free‐living, heterotrophic bacteria rather than soil crusts play an important role in biological N_2_ fixation in desert soil profiles.

### 4.2 Microbial Response to Excess C and Its Relationship With N_2_ Fixation

In this study, increased CO_2_ efflux in both soils with positive rhizosphere effects after adding excess carbon (Figure 3) and its positive correlation with some microbial communities (Table 4) suggest that the soil priming effect is likely caused by rhizosphere‐specific microbial communities. Moreover, the significant positive correlation between soil CO_2_ emission and AN, ${\mathrm{NO}}_{3}^{-}$‐N, and soil δ^15^N values (Table 4) indicates a close link between SOC mineralization and N fixation. Excess glucose provides a sufficient energy and carbon source for microorganisms. Also, the assimilation of carbon and nitrogen by microorganisms is synchronized (Cui et al. 2020). As a result, the microorganisms mineralizing SOC need a continuous supply of available N while sequestering carbon. At this point, the N‐fixing microorganisms that have access to carbon sources will function (Hoogmoed et al. 2014). Therefore, the biological N_2_ fixation in the desert soil likely involves a complex process of C‐ and N‐fixing by multi‐microbial participation.

Consistent increases in the relative abundance of some microorganisms (e.g., Halomonadaceae, Promicromonosporaceae, and Micrococcaceae) and CO_2_ efflux in both soils after adding carbon (Figures 3 and 5) suggest that these microorganisms might be primarily involved in the mineralization of SOC (Jiang et al. 2014). However, distinct responses of other microorganisms (e.g., Bacillaceae, Nocardiopsaceae, and Paenibacillaceae) in two soils to excess carbon addition may correspond to the strong contrast of microbial N fixation between them. Significant correlations between N fixation and certain microbial genera (e.g., *Gillisia*, *salinimicrobium*, and *Paraliobacillus*) were also found in this study. These specific microorganisms, some of which act as free‐living N_2_‐fixing bacteria, have been reported in previous studies (Li et al. 1992; Merzaeva and Shirokikh 2006; Eskin 2012).

N fixation in soils is accomplished by a catalytic protein—nitrogenase. The Mo‐dependent nitrogenase (mainly encoded by the *nifH* gene) is the only nitrogenase that forms symbiotic N fixation between diazotrophs and vascular plants (Bellenger et al. 2020). However, in addition to typical molybdenum dinitride enzymes, there are two known alternative nitrogenases that can fix N by substituting vanadium (V) or iron (Fe) for molybdenum dinitride (Eady 1988; Thiel and Pratte 2013). They have also been identified as ecologically important populations, including free‐living bacteria in soils and fresh water. In the current study, although *nifH* gene abundance and soil δ^15^N values showed a significantly positive correlation, their responses to excess carbon addition were not consistent (Tables 3 and 4 and Figure 2). Moreover, excess carbon addition had no significant effect on gene abundance (Table S1). This suggests that *nifH* gene abundance does not fully reflect microbial N fixation in soils. In other words, the *nifH* gene is probably not the only microbial N fixation gene in this study area.

### 4.3 Factors Driving for N_2_ Fixation

Salinity usually has an adverse effect on biological N_2_ fixation (Delgado et al. 1994). In arid desert regions; however, salinity may be a critical factor influencing N fixation and plant adaptation, as highlighted by Li et al. (2021). The research has shown that the symbiotic N fixation of a desert legume *Alhagi sparsifolia* is tolerant to salt stress and can be adjusted in response to variation in N availability and soil salinity. In the current study, compared to the sandy soil, the heavy‐textured soil with significantly higher salinity levels (Table 2) exhibited greater N fixation with significant positive rhizosphere effects (Figure 2), which is generally consistent with the above observation. Such relationships between salt tolerance and biological N_2_ fixation suggest that desert shrubs are likely to increase soil nutrient availability and enhance their adaptation to saline environments through root‐associated microorganisms.

Furthermore, the addition of organic matter can effectively stimulate free‐living N_2_ fixation (Reed et al. 2011). Under excess carbon addition, heavy‐textured soils released more CO_2_ than sandy soils (Figure 3), revealing higher levels of microbial biomass and activity in heavy‐textured soils (Aira and Domínguez 2010). Meanwhile, significant N_2_ fixation was also found throughout the entire profile of heavy‐textured soils, whereas in sandy soils, only the bulk topsoil showed slight N_2_ fixation (Figure 2). This suggests that the heavy‐textured soils have a greater capacity for free‐living N_2_ fixation under N deficiency compared with sandy soils.

The occurrence of biological N_2_ fixation is constrained by soil texture (Warren Raffa et al. 2015), given the content of SOC and nutrients varies depending on the soil texture (Dupuis and Whalen 2007). In the current study, some soil properties (i.e., AN, ${\mathrm{NO}}_{3}^{-}$‐N, TP, SOC, TN, and clay content) showed positive and significant correlations with N_2_ fixation in soils. Heavy‐textured soils with higher nutrient content (Table 2) create favorable conditions for the colonization of N‐fixing bacteria. Hence, biological N_2_ fixation is more likely to occur under N‐deficient conditions. In contrast, sandy soils with a high proportion of sand particles result in poor water and nutrient retention capacity (Tables 1 and 2). This makes it difficult for N‐fixing bacteria to establish colonies. These factors may explain the distinct responses of N_2_ fixation to excess carbon source observed in the two soils.

Furthermore, significant positive rhizosphere effects on N_2_ fixation found in the heavy‐textured soil (Figure 2) may be attributed to the recruitment and selection of beneficial microorganisms (e.g., N fixers) by specific organic compounds secreted by plant roots. Moreover, the heavy‐textured soil also showed a similar microbial community structure between bulk and rhizosphere soils under N stress, compared to the sandy soil (Figure 6). These results suggest that heavy‐textured soils with specific microbial communities are more closely related to plants than sandy soils in terms of N_2_ fixation. For sandy soils, the corresponding shrub communities may absorb N from groundwater or meltwater due to their well‐developed deep root systems (Dai et al. 2015). The N fixation by plant endophytes may also be an important pathway (Bokhari et al. 2019). Therefore, in desert ecosystems, especially in sandy soils, there remains much uncertainty regarding N use by plants, which may involve a variety of N fixation pathways.

The processes and mechanisms involved in biological nitrogen fixation are complex and numerous, especially in the case of free‐living N_2_ fixation, which is still poorly understood. Our results are obtained from a limited study area. Further work is needed to determine the applicability of the conclusions for different sites in desert regions. Meanwhile, the identification of specific members of the N‐fixing communities through incorporating culture‐based approaches is also our next research focus.

## 5 Conclusion

In this study, microbial N fixation in two desert soils with contrasting textures was estimated through soil culture experiments with ^15^N‐labeled N_2_. The soil δ^15^N values in N‐deficient soils created by excess glucose addition showed that N fixation was significant throughout the profile (0–60 cm) of the heavy‐textured soil. However, only the bulk topsoil (0–20 cm) showed slight N fixation in the sandy soil. The heavy‐textured rhizosphere soil exhibited the greatest N fixation and microbial activity. Compared to the sandy soil, the microbial community structure in the heavy‐textured soil exhibited greater similarity across soil depths, bulk and rhizosphere soils, implying a closer relationship between soil and plant. We found significantly positive correlations between N fixation and some soil properties (e.g., TN, AN, ${\mathrm{NO}}_{3}^{-}$‐N, TP, SOC, and clay content), CO_2_ efflux, *nifH* gene abundance, and the relative abundance of certain microbial genera (e.g., *Gillisia*, *Salinimicrobium*, *Paraliobacillus*, *Palleronia*, and *Isoptericola*). The distinct responses of microbial N fixation observed in the two soils are likely a result of differences in nutrient content caused by soil texture. These findings provide insights into the mechanisms underlying microbial N fixation in desert soil profiles.

## Author Contributions

**Chenhua Li:** data curation (lead), methodology (equal), writing – original draft (lead). **Ran Liu:** investigation (equal), supervision (equal), writing – review and editing (equal). **Lisong Tang:** conceptualization (equal), writing – review and editing (equal). **Li Jiang:** formal analysis (equal), project administration (equal).

## Conflicts of Interest

The authors declare no conflicts of interest.

## Supporting information


**Table S1.** Effects of carbon addition and soil texture on the soil parameters and the relative abundance of main groups at the phylum level. Results from three‐way ANOVA testing at *p* < 0.05.
**Table S2.** Forward selection of soil variables [i.e., soil textures (silt, clay, and sand), electrical conductivity (EC), organic carbon (SOC), total nitrogen (TN), and total phosphorus (TP)] with significant effects on soil microbial communities under excess glucose addition by redundancy analysis with Monte Carlo test.

## Data Availability

The raw sequences were submitted to the NCBI Sequence Read Archive database under the BioProject PRJNA1169205 (http://www.ncbi.nlm.nih.gov/bioproject/1169205).
