# Supplementary material for: Heavy‐Textured Rhizosphere Soils Enhance Microbial Nitrogen Fixation in a Desert Shrub Ecosystem
Source: Ecol Evol. 2025 Apr 9;15(4):e71210. doi: 10.1002/ece3.71210 (PMC11981880; doi:10.1002/ece3.71210)
Supplement: Supplementary file 1 — Table S1. Effects of carbon addition and soil texture on the soil parameters and the relative abundance of main groups at the phylum level. Results from three‐way ANOVA testing at p < 0.05. Table S2. Forward selection of soil variables [i.e., soil textures (silt, clay, and sand), electrical conductivity (EC), organic carbon (SOC), total nitrogen (TN), and total phosphorus (TP)] with significant effects on soil microbial communities under excess glucose addition by redundancy analysis with Monte Carlo test. [file ECE3-15-e71210-s001.docx]

**Table S1**

Effects of carbon addition and soil texture on the soil parameters and the relative abundance of main groups at phylum level. Results from three-way ANOVA testing at *p*<0.05

|  | Depth (D) | |  | Carbon addition (C) | |  | Soil texture (S) | |  | D ×C | |
| --- | --- | --- | --- | --- | --- | --- | --- | --- | --- | --- | --- |
|  | *F* | *p* |  | *F* | *p* |  | *F* | *p* |  | *F* | *p* |
| δ^15^N | 31.81 | <0.001 |  | 49.66 | <0.001 |  | 41.94 | <0.001 |  | 23.07 | <0.001 |
| CO_2_ | 10.95 | 0.003 |  | 43.38 | <0.001 |  | 7.20 | 0.015 |  | 6.51 | 0.017 |
| nifH | 22.24 | <0.001 |  | 1.25 | 0.270 |  | 17. 69 | <0.001 |  | 0.15 | 0.859 |
| Actinobacteria | 61.10 | <0.001 |  | 15.27 | <0.001 |  | 20.68 | <0.001 |  | 5.52 | 0.036 |
| Proteobacteria | 78.42 | <0.001 |  | 30.76 | <0.001 |  | 86.01 | <0.001 |  | 30.93 | <0.001 |
| Firmicutes | 152.00 | <0.001 |  | 279.81 | <0.001 |  | 34.59 | <0.001 |  | 29.03 | <0.001 |
| Bacteroidetes | 12.26 | 0.001 |  | 33.92 | <0.001 |  | 19.99 | <0.001 |  | 1.15 | 0.324 |
| Chloroflexi | 200.79 | <0.001 |  | 284.72 | <0.001 |  | 111.61 | <0.001 |  | 16.13 | <0.001 |
| Gemmatimomatetes | 11.12 | 0.002 |  | 254.73 | <0.001 |  | 27.98 | <0.001 |  | 0.37 | 0.690 |
|  |  |  |  |  |  |  |  |  |  |  |  |
|  | D × S | |  | C × S | |  | D ×C × S | |  |  | |
|  | *F* | *p* |  | *F* | *p* |  | *F* | *p* |  |  |  |
| δ^15^N | 21.35 | <0.001 |  | 2.89 | 0.069 |  | 2.92 | 0.069 |  |  |  |
| CO_2_ | 1.80 | 0.119 |  | 6.09 | 0.017 |  | 1.77 | 0.126 |  |  |  |
| nifH | 5.62 | 0.031 |  | 2.46 | 0.074 |  | 1.75 | 0.129 |  |  |  |
| Actinobacteria | 16.24 | <0 .001 |  | 3.98 | 0.051 |  | 3.93 | 0.053 |  |  |  |
| Proteobacteria | 10.06 | 0.004 |  | 15.63 | <0.001 |  | 7.35 | 0.023 |  |  |  |
| Firmicutes | 33.35 | <0.001 |  | 22.54 | <0.001 |  | 9.32 | 0.006 |  |  |  |
| Bacteroidetes | 3.61 | 0.058 |  | 9.33 | 0.006 |  | 1.75 | 0.129 |  |  |  |
| Chloroflexi | 38.45 | <0.001 |  | 3.91 | 0.052 |  | 3.56 | 0.064 |  |  |  |
| Gemmatimomatetes | 23.86 | <0.001 |  | 3.88 | 0.059 |  | 3.93 | 0.052 |  |  |  |

*******, *p* < 0.001; ******, *p* < 0.01; *****, *p* < 0.05

**Table S2**

Forward selection of soil variables [i.e. soil textures (silt, clay, and sand), electrical conductivity (EC), organic carbon (SOC), total nitrogen (TN), and total phosphorus (TP)] with significant effects on soil microbial communities under excess glucose addition by redundancy analysis with Monte Carlo test

|  | Silt | Clay | Sand | EC | SOC | TN | TP |
| --- | --- | --- | --- | --- | --- | --- | --- |
| Rank | 1 | 2 | 3 | 4 | 5 | 6 | 7 |
| *F* | 11.716 | 11.569 | 10.230 | 9.895 | 6.212 | 5.510 | 5.004 |
| *P* | 0.002^**^ | 0.002^**^ | 0.004^**^ | 0.004^**^ | 0.006^**^ | 0.006^**^ | 0.012^*^ |

******, *p* < 0.01; *****, *p* < 0.05
